# Segmentation precedes face categorization under suboptimal conditions

**DOI:** 10.3389/fpsyg.2015.00667

**Published:** 2015-05-26

**Authors:** Carlijn Van Den Boomen, Johannes J. Fahrenfort, Tineke M. Snijders, Chantal Kemner

**Affiliations:** ^1^Department of Experimental Psychology, Helmholtz Institute, Utrecht UniversityUtrecht, Netherlands; ^2^Department of Developmental Psychology, Utrecht UniversityUtrecht, Netherlands; ^3^Department of Cognitive Psychology, Vrije UniversiteitAmsterdam, Netherlands; ^4^Donders Institute for Brain, Cognition and Behaviour, Centre for Cognitive Neuroimaging, Radboud University NijmegenNijmegen, Netherlands; ^5^Centre for Language Studies, Radboud University NijmegenNijmegen, Netherlands; ^6^Department of Child and Adolescent Psychiatry, Rudolf Magnus Institute of Neuroscience, University Medical CenterUtrecht, Netherlands

**Keywords:** EEG, face processing, visual system, low-level vision, high-level vision, categorization

## Abstract

Both categorization and segmentation processes play a crucial role in face perception. However, the functional relation between these subprocesses is currently unclear. The present study investigates the temporal relation between segmentation-related and category-selective responses in the brain, using electroencephalography (EEG). Surface segmentation and category content were both manipulated using texture-defined objects, including faces. This allowed us to study brain activity related to segmentation and to categorization. In the main experiment, participants viewed texture-defined objects for a duration of 800 ms. EEG results revealed that segmentation-related responses precede category-selective responses. Three additional experiments revealed that the presence and timing of categorization depends on stimulus properties and presentation duration. Photographic objects were presented for a long and short (92 ms) duration and evoked fast category-selective responses in both cases. On the other hand, presentation of texture-defined objects for a short duration only evoked segmentation-related but no category-selective responses. Category-selective responses were much slower when evoked by texture-defined than by photographic objects. We suggest that in case of categorization of objects under suboptimal conditions, such as when low-level stimulus properties are not sufficient for fast object categorization, segmentation facilitates the slower categorization process.

## Introduction

Faces contain information of high ecological value: their presence and content hold important cues for behavior toward another person and the environment. Faces are often considered a special category of objects, because they evoke activity in a specific brain area [the fusiform face area (FFA)] and because humans seem particularly sensitive to them (McKone and Robbins, 2011, but see Gauthier et al., 1999). However, even though processing of faces in the brain has been studied for many years, it is not yet fully clear how subprocesses of face processing relate to each other. Two subprocesses that are essential for face perception are categorization and segmentation. Face categorization involves the process of placing an object in the specific category ‘faces’ and not in another one, such as ‘houses’ (Kanwisher et al., 1997; Epstein and Kanwisher, 1998). There is increasing evidence that successful categorization of a face in the brain is not sufficient for face perception. Additional processes are required, such as (face) segmentation, which includes integration of local visual elements into a face or other object, and segregation of the face from its background (Lamme and Roelfsema, 2000; Hochstein and Ahissar, 2002; Fahrenfort et al., 2012). The temporal relation between category-selective and segmentation-related responses in the brain is currently unclear. Increased insight in the temporal relation is crucial to understand face processing in typical adults, but also in populations in which face processing has developed abnormally, since manipulations or abnormalities in the first process could affect the subsequent one as well. The current study investigates the temporal relation between face category-selective and segmentation-related responses in the adult brain.

**FIGURE 1:**
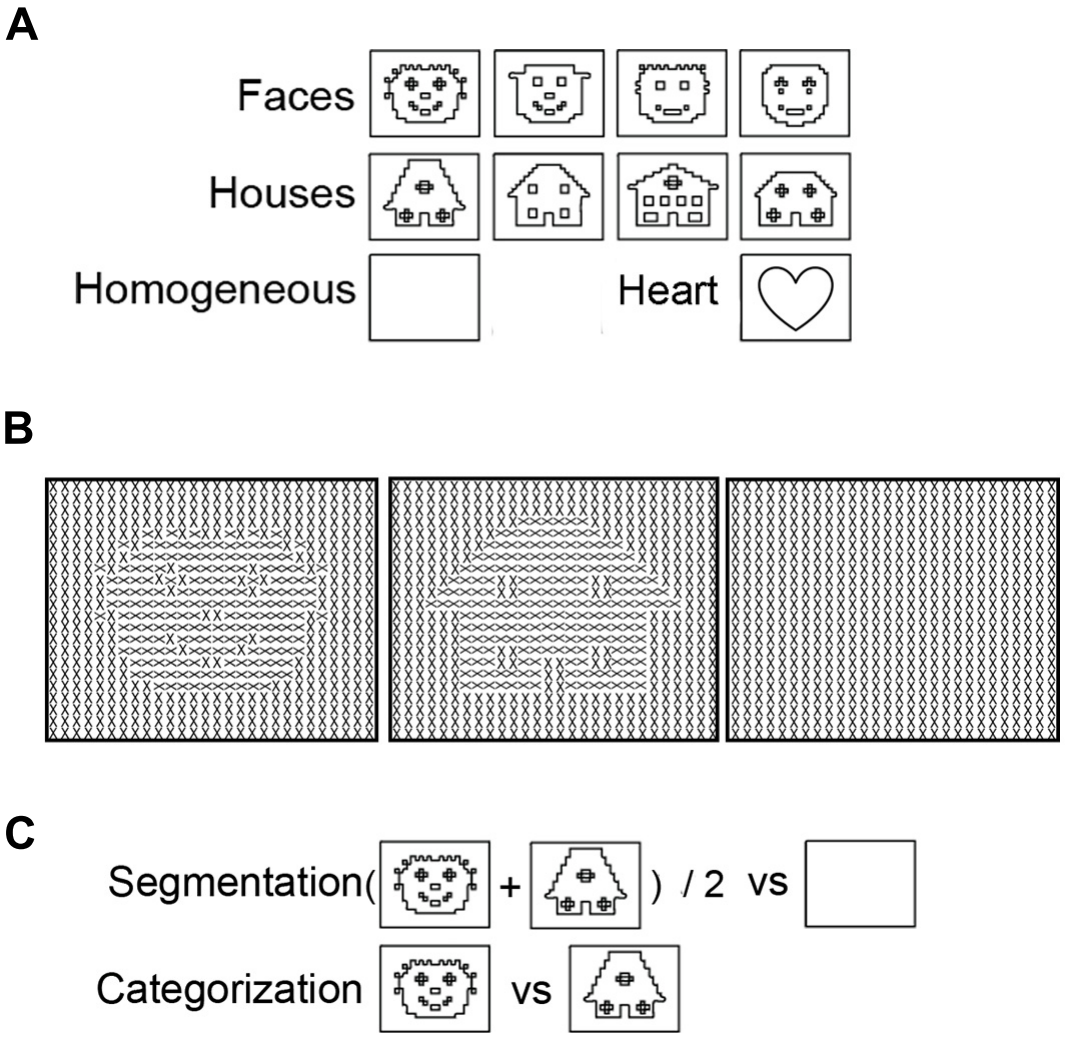
**Examples of stimuli presented to study segmentation and categorization of texture-defined objects. (A)** Schematic versions of the images of faces, houses, and homogeneous textures. Black lines represent object borders, defined by differences in Gabor orientation. **(B)** Schematic versions of the stimuli. For clarity purposes, black lines instead of Gabor patches are depicted in this figure. **(C)** Contrasts to separate Segmentation-related responses ((face+house)/2 versus homogeneous) and Categorization-selective responses (face versus house).

Early theories suggested that segmentation precedes categorization (Rubin, 1915/1958). On the other hand, more recent theories and behavioral studies suggest that segmentation might follow categorization (Peterson, 1994), or that both processes occur interactively or in parallel (Vecera and O’Reilly, 1998; Hochstein and Ahissar, 2002). Although many studies have been conducted, this issue is not resolved. In behavioral studies, manipulations of stimulus presentation settings led to varying behavioral results that supported either one of these theories (Peterson, 1993, 1994; Grill-Spector and Kanwisher, 2005; Mack et al., 2008; Wyatte et al., 2012). A problem with neurophysiological studies is that they typically use stimuli that do not allow studying these processes separately. Instead, they use stimuli that allow for either segmentation or categorization contrasts. Segmentation (usually of abstract stimuli) is often studied by comparing brain activity evoked by stimuli containing multiple (line) elements that either all have the same orientation (referred to as homogeneous stimulus; **Figure 1**) or together form a figure on a background (texture-defined or figure stimulus; e.g., Bach and Meigen, 1992, 1998; Lamme et al., 1992; Caputo and Casco, 1999; Scholte et al., 2008). Face categorization on the other hand is studied by comparing brain activity evoked by faces and other objects, such as houses (e.g., Eimer, 2000b). However, presentation of natural stimuli makes it hard to study categorization and segmentation processes in a single experiment. To study segmentation processes, one could create a homogeneous version of photographic objects by block- or phase-scrambling the intact object (e.g., Malach et al., 1995; Grill-Spector et al., 2000). The resulting stimuli do, however, not perfectly balance stimulus properties between object and homogeneous conditions in a way that classic segmentation studies do (e.g., Lamme, 1995; Lamme et al., 1998). Consequently, studies using scrambling techniques find correlates of object segmentation in a region called the lateral occipital cortex higher up the visual hierarchy, while classic segmentation studies identify feedback processes to lower visual areas.

A recent stimulus design makes it possible to study categorization and segmentation-related responses at the same time (Fahrenfort et al., 2012). In so-called texture-defined objects, the presence of texture and category content (i.e., being a face or a house; **Figure 1**) can be manipulated while controlling for low-level visual stimulation. These stimuli contain multiple Gabor elements that form a homogeneous or figure stimulus, in which the figure is a face or house. Functional magnetic resonance imaging (fMRI) responses evoked by texture-defined objects showed that categorization- can occur independently of segmentation-related responses: faces activated the FFA, even in the absence of sustained segmentation-related responses (Fahrenfort et al., 2012). This study showed that presentation of texture-defined objects is a promising design to investigate segmentation-related and category-selective responses and further explore their interrelation.

The current study investigated the temporal relation between segmentation-related and category-selective responses evoked by texture-defined objects, recorded using electroencephalography (EEG). The high temporal resolution of EEG makes it possible to determine the timing and temporal relation of these processes. This was carried out by contrasting brain activity evoked by faces versus houses (category-selective response) and evoked by objects versus homogeneous stimuli (segmentation-related response). We were particularly interested in face categorization because faces hold important social information, but are processed differently in various populations (e.g., Golarai et al., 2006). As such, this study increases our understanding of typical social information processing, and forms a background for future research into atypical face processing. Contrasting face to house processing is common in studies on category-selective responses, especially when investigating neural responses to stimuli presented under suboptimal conditions (Tong et al., 1998; Yantis and Serences, 2003). We focused on segmentation-related and category-selective responses at any timepoint until 500 ms after stimulus presentation. Analyses were not restricted to the N170 peak, because previous research suggests that category-selective responses might not selectively occur at this peak (Thierry et al., 2007). Based on behavioral studies, any temporal relation between segmentation-related and category-selective responses could be hypothesized (Peterson, 1993, 1994; Grill-Spector and Kanwisher, 2005; Mack et al., 2008; Wyatte et al., 2012). However, the fMRI results by Fahrenfort et al. (2012) revealed that category-selective responses occurred independently of segmentation-related responses when evoked by texture-defined objects. Because in that study categorization occurred in absence of segmentation, we hypothesized that categorization- would temporally precede segmentation-related responses. This would be visible in an earlier peak in category-selective responses than in segmentation-related responses in the EEG signal.

The current article presents four EEG experiments (**Table 1**). First, the experiment that led to the main outcome, i.e., the temporal relation between segmentation-related and category-selective responses. This was supplemented with a behavioral control study to study whether texture-defined objects could be behaviorally categorized. After this, we describe three additional experiments to study the effect of presentation duration and the difference between photographic and texture-defined objects in an attempt to resolve contradictory outcomes of studies using variable stimulus material (Peterson, 1993, 1994; Grill-Spector and Kanwisher, 2005; Mack et al., 2008; Wyatte et al., 2012).

**Table 1:** Overview of methods and results of Experiments 1-4

Experiment	Participants # (age, SE)	Stimulus duration	Stimuli	Contrasts^4^	Results
(1) Texture-defined objects - long presentation duration	23 (21.4; 0.4)^A^	800 ms	Texture-defined faces, houses, and homogeneous	Categorization and segmentation	Segmentation-related precedes slow category-selective responses
**Additional experiments**
(2) Photographic objects - long presentation duration	23 (21.4; 0.4)^A^	800ms	Photographic faces and houses	Categorization	Fast category-selective responses
(3) Photographic objects - short presentation duration	18 (22.1; 0.5)^B^	92ms	Photographic faces and houses	Categorization	Fast category-selective responses
(4) Texture-defined objects - short presentation duration	18 (22.1; 0.5)^B^	92ms	Texture-defined faces, houses and homogeneous (dichoptic presentation) - Visible and invisible	Categorization and segmentation	Only segmentation-related but no category-selective responses in visible condition

^A,B^A or B indicate the same group of participants (group A or B).

^*^Categorization = face-house; Segmentation = object ((face+house)/2) - homogeneous.

## Experiment 1: Segmentation Precedes Categorization of Texture-Defined Objects

The first experiment investigated the presence of, and temporal relation between segmentation-related responses (i.e., difference in activity evoked by faces and houses versus homogeneous images) and category-selective responses (i.e., difference in activity evoked by faces versus houses). For this purpose, we presented texture-defined stimuli that consisted of multiple Gabor elements together forming a face, house, or homogeneous image.

### Methods

#### Participants

Twenty-three healthy adults (average age 21.4 years, SD = 1.9; 12 males) participated in the study. Two other participants were excluded from the analyses, due to technical or human error during data acquisition. All participants had normal or corrected-to-normal vision and did not suffer from any psychiatric disorder. The research meets all applicable standards for ethics of experimentation and research integrity. The Medical Ethics Committee of the University Medical Center Utrecht approved all experiments. Participants provided written informed consent prior to participation, according to the Declaration of Helsinki (World Medical Association, 2013). Participants received monetary reward, or participation points (as part of the Bachelor’s curriculum in Psychology). Data reported in all experiments can be accessed via a request to the data handling committee. Please contact the corresponding author for details.

#### Procedure and Stimulation

Electroencephalography was acquired during presentation of texture-defined stimuli, in a luminance-controlled lab. This run contained three stimulus conditions: faces, houses, and homogeneous (**Figure 1A**). All stimuli contained crosses, created by two superimposed Gabor patches (**Figure 1B**). In the face- and house-stimuli, orientation of crosses differed between fore- and background. Homogeneous stimuli contained crosses with the same orientation. The orientation of crosses was counterbalanced within conditions, such that each orientation occurred equally often at each part of the visual field. There were 64 trials per stimulus condition, presented in a randomized order. Six percent of the stimuli consisted of a heart created using a similar matrix of Gabor elements as the texture-defined stimuli (**Figure 1A**). A stimulus of a heart was chosen because of the absence of facial (e.g., eyes, mouth) or house-like features. Participants had to press the spacebar when a heart-stimulus was present. With this task, we intended to attract and maintain participants’ attention to the screen while minimizing task-induced attentional differences between conditions. The presentation sequence contained target presentation for 800 ms, followed by a mask for 50 ms preventing possible retinal after-effects, and an inter-stimulus interval of 1600–2000 ms. The mask consisted of a field of Gabor elements with random orientation and the inter-stimulus interval contained a gray screen with a fixation cross. Participants used a chinrest to stay at a distance of 45 cm from the screen, such that stimuli measured 16.9∘ × 12.7∘ of visual angle.

#### EEG Recording and Analyses

##### Recording

A Biosemi Active Two EEG system (Biosemi, Amsterdam, The Netherlands) recorded EEG activity from 32 electrodes. We positioned electrodes at standard EEG recording locations according to the international 10/20 system. Electrodes above and below the left eye recorded vertical EOG to detect blinks, and electrodes near the outer canthi of the eyes recorded horizontal EOG to detect horizontal eye movements. Two additional electrodes were placed at the left and right mastoid to maintain the possibility of oﬄine re-referencing to these electrodes. During recording, the EEG sampling rate was 2048 Hz. Two electrodes in the cap, the CMS (Common Mode Sense) and DRL (Driven Right Leg), provided an ‘active ground.’

##### Preprocessing analyses

Preprocessing analyses were performed in Brain Vision Analyzer (Amsterdam, the Netherlands). First, we resampled data oﬄine to 512 Hz, and filtered them with a high-pass filter of 0.1592 Hz (24 dB/oct), a low-pass filter of 20 Hz (24 dB/oct) and a notch filter of 50 Hz. In order to compare event related potentials (ERPs), epochs of 100 ms pre-stimulus (baseline) until 800 ms post-stimulus were extracted from the continuous data. Epochs with large artifacts were removed. Activity was an artifact when amplitudes were below -200 or above 200 μV. A regression analysis based on eye-movements detected by vertical EOG (blinks) and horizontal EOG electrodes (horizontal eye-movements) removed ocular artifacts from the EEG (Gratton et al., 1983). Then, additional artifacts were rejected for each individual electrode. Activity was an artifact when there was a voltage change of 50 μV per sampling point, a difference of 1 μV per 100 ms, or amplitudes below -50 or above 50 μV. Activity was re-referenced to the average of all 32 cap-electrodes. We corrected for baseline activity, with baseline defined from -100 ms. to stimulus onset. Finally, data was averaged per condition.

##### Cluster-based permutation analyses

Before analyzing the temporal relation between segmentation-related and category-selective responses using ERP peak analyses, we used cluster-based permutation tests to study whether and in which cluster of electrodes such responses were evoked (see Maris and Oostenveld, 2007 for a detailed description of the methods and, e.g., Snijders et al., 2007; Rousselet et al., 2010; Fahrenfort et al., 2012 for examples of previous EEG studies using this method). The cluster-based permutation test was performed in Fieldtrip^1^. This test effectively controls the multiple comparison problem [in this case 9 electrodes (parieto-occipital) × 256 timepoints]. In a first step all electrode-timepoint-combinations are identified in which the *t*-statistic for the EEG amplitude difference between two conditions reaches a specific threshold (*p* < 0.05). In a second step all electrode-timepoint-combinations that are connected spatially (adjacent electrodes) or temporally (adjacent timepoints) are clustered. The sum of the *t*-values of all electrode-time-point combinations within each cluster is calculated. Then, using a permutation test (randomizing the assignment of conditions) a distribution of the summed cluster-value is made under the null-hypothesis of no effect. The *p*-value of the observed cluster specifies the probability of observing such a large summed cluster-value when there is actually no effect. The cluster-based permutation test is non-parametric and corrects for multiple comparisons, as all timepoints and electrodes are assessed in one single test. As such, it allowed studying whether segmentation-related and category-selective responses were present in any of the occipital or parietal electrodes at any timepoint between 0 and 500 ms after stimulus onset, with a minimized risk of false alarms (FAs). To determine segmentation-related responses, we compared responses evoked by texture-defined objects (faces and houses) to those evoked by homogeneous images. Contrasting responses evoked by face versus house images resulted in isolation of category-selective responses (**Figure 1C**).

While cluster-based permutation tests are very effective in establishing whether an effect is present while controlling the number of comparisons (as only a single test is used), cluster-based permutation tests are not well-suited to determine whether the conditions differ at a specific timepoint or electrodes. We used ERP peak analyses, which are specifically sensitive to latency differences, to complement cluster-based permutation tests and reveal whether segmentation-related responses show a maximal response earlier in time than category-selective responses.

##### ERP peak analyses

**FIGURE 2:**
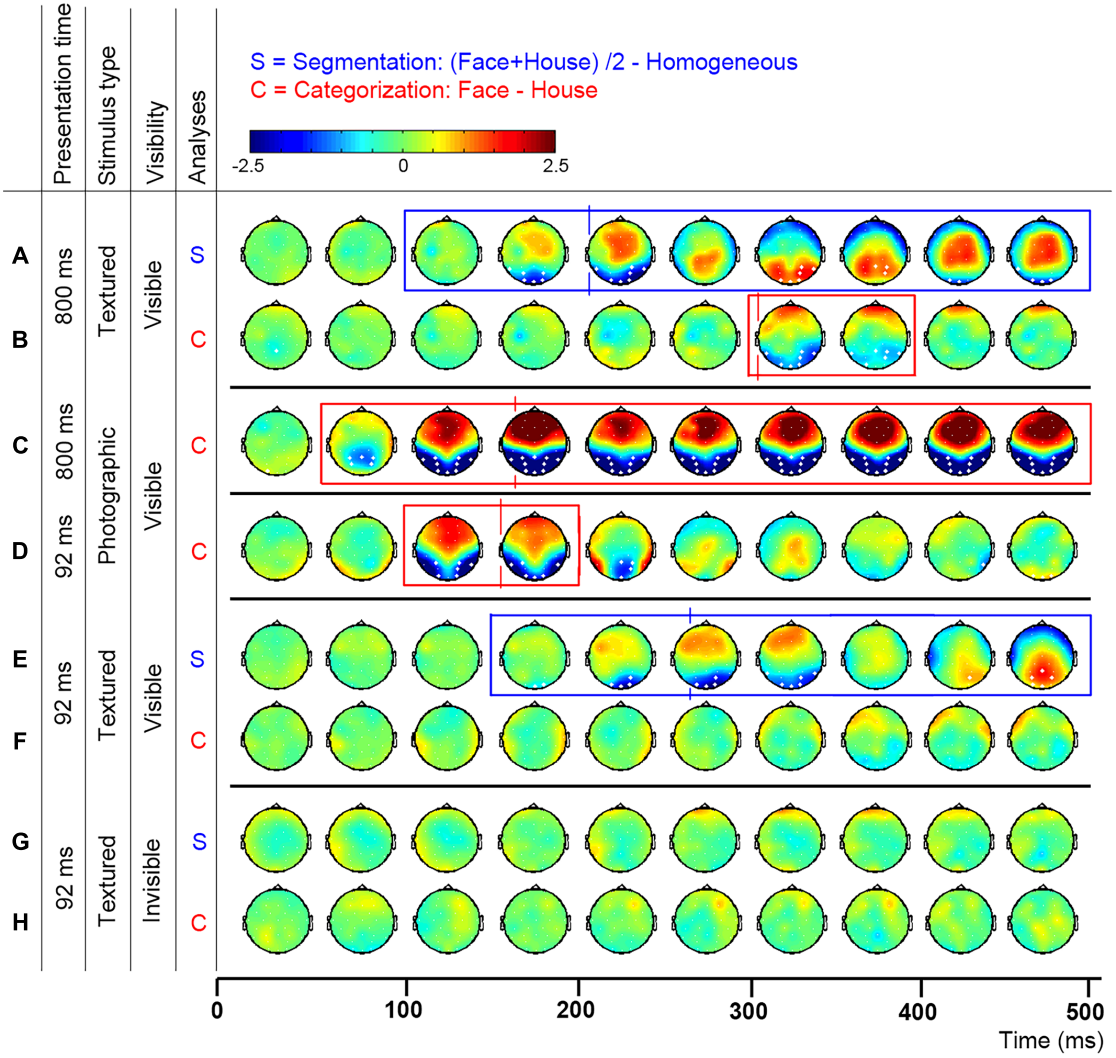
**Topographic distribution of evoked activity in time-bins of 50 ms, resulting from segmentation-related (S) and category-selective (C) contrasts**. Thick white electrodes indicate cluster-corrected significant sites. Rectangles indicate cluster-corrected significant time-bins. Vertical lines indicate the timepoint at which activity was maximal, revealed by ERP peak-detection of segmentation-related activity at the Oz and category-selective responses at the P8 electrode. Activity was evoked by various stimulus and presentation settings (described on the left): Experiment 1, texture-defined objects presented for 800 ms evoked segmentation-related activity **(A)** and category-selective responses **(B)**; Experiment 2, photographic objects presented for 800 ms evoked category-selective responses **(C)**; Experiment 3, photographic objects presented for 92 ms evoked category-selective responses **(D)**; Experiment 4, texture-defined objects presented for 92 ms evoked in the visible condition segmentation-related activity **(E)** but no category-selective responses **(F)**, and in the invisible condition no segmentation-related activity **(G)** or category-selective responses **(H)**.

If the cluster-based permutation analyses revealed significant segmentation or category-selective responses, we used ERP peak latency analyses to reveal the timing of this response. Comparison of the timing of segmentation-related to category-selective responses revealed their temporal relation. To perform these analyses, we first contrasted activity evoked by object versus homogeneous-stimuli and by face- versus house-stimuli. This created segmentation- and categorization- difference waves. Then, peaks were detected as the local maximum of activity in each difference wave between 0 and 500 ms after stimulus onset. Electrodes of interest for peak analyses (Oz, P8, and P7) were selected based on previous research and results of the cluster-based permutation tests. Segmentation based on low-level stimulus properties is typically studied in the Oz electrode (Bach and Meigen, 1992; Lamme et al., 1992). However, segmentation-related responses could occur at other electrodes as well, either as an effect of volume conduction, or possibly reflecting responses of higher-level visual areas to the presence of a figure. This activity is important to analyze in order to exclude the possibility that earlier segmentation-related than category-selective responses reflect a typical feedforward flow of information (reflected in earlier peaks a early Oz than later P8 electrodes). Therefore, segmentation-related responses were studied at both Oz and the P8 electrode. Face category-selective responses are typically maximal over the occipito-temporal P8 electrode (Eimer, 2000a). We studied face category-selective responses in both P8 and P7 to control for laterality effects (Rossion and Jacques, 2008). Cluster-based permutation analyses of the current data confirmed segmentation-related responses over the Oz-electrode and category-selective responses over the P7 and P8 electrodes (**Figure 2**). Note that other electrodes, over which responses were significantly present as well, showed the same pattern of results as described below.

### Results and Discussion

**Figures 2A,B** show the activity resulting from two contrasts: evoked by object versus homogeneous (to detect segmentation-related responses, **Figure 2A**) and by face versus house stimuli (category-selective responses, **Figure 2B**). Boxes represent the interval over which cluster-based permutation analyses detected the response. Vertical lines show the time at which ERP peak analyses revealed that the response was maximal. Blue represents significant segmentation-related, and red represents category-selective responses.

#### Cluster-based permutation analyses

Texture-defined stimuli evoked both segmentation-related and category-selective responses (**Figures 2A,B**). Segmentation contrasts (i.e., object > homogeneous) evoked three clusters: clusters between 135 and 283 ms (negative cluster, i.e., activity evoked by object is more negative than by homogeneous stimuli), 242 and 424 ms (positive) and 414 and 500 ms (negative) after stimulus onset. Categorization contrasts (i.e., face > house) evoked one cluster: between 289 and 416 ms (negative cluster, i.e., activity evoked by face is more negative than by house stimuli).

#### ERP peak analyses

**FIGURE 3:**
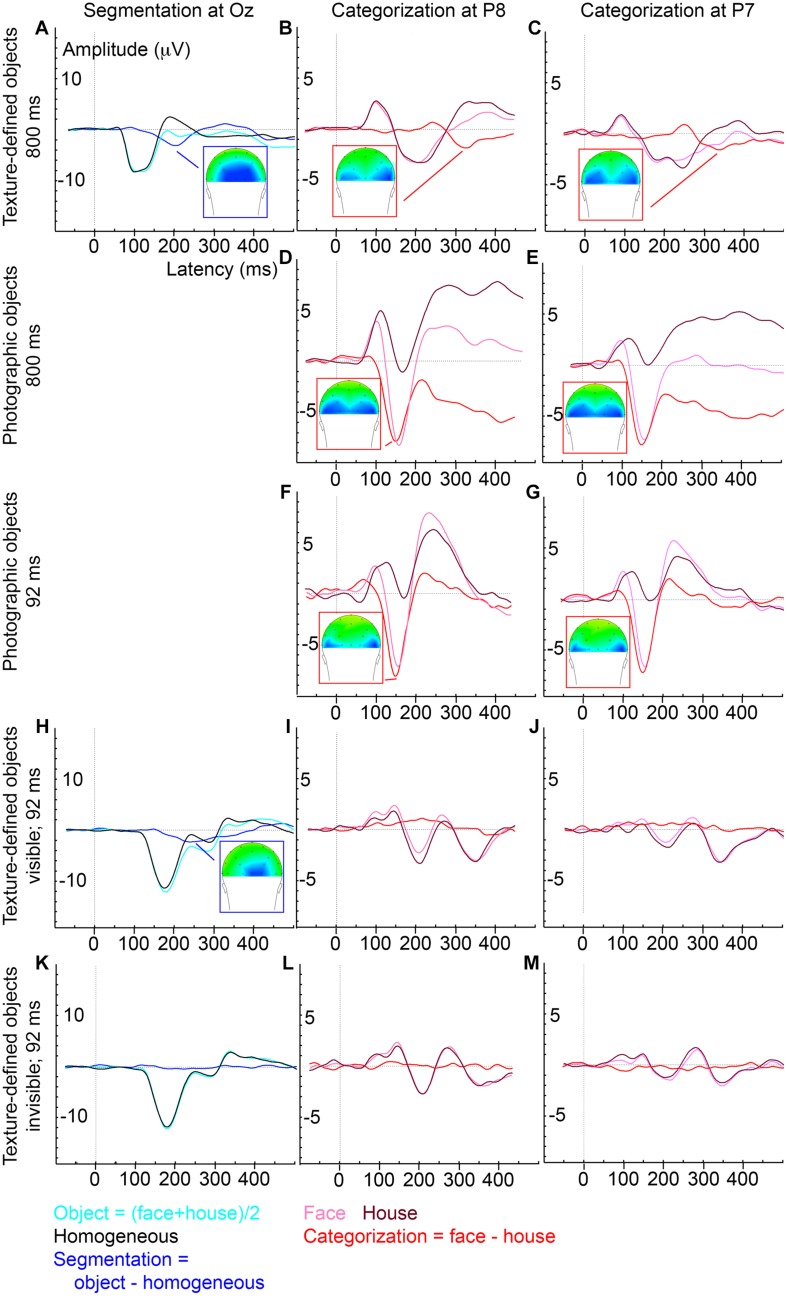
**Average ERP evoked by various stimulus and presentation settings (described on the left)**. Experiment 1, texture-defined objects presented for 800 ms, evoked segmentation-related activity **(A)** and category-selective responses at P8 **(B)** and P7 **(C)**; Experiment 2, photographic objects presented for 800 ms, evoked category-selective responses at P8 **(D)** and P7 **(E)**; Experiment 3, photographic objects presented for 92 ms, evoked category-selective responses at P8 **(F)** and P7 **(G)**; Experiment 4, texture-defined objects presented for 92 ms evoked in the visible condition segmentation-related activity **(H)** but no category-selective responses at P8 **(I)** or P7 **(J)**, and in the invisible condition no segmentation-related activity **(K)** or category-selective responses at P8 **(L)** or P7 **(M)**.

ERP peak analyses revealed that segmentation-related activity precedes category-selective responses. Peak detection showed that segmentation-related responses were maximal at 209 ms (SE = 5.9; **Figure 3A**). Category-selective responses were maximal at 304 ms (SE = 18.8) after stimulus onset at P8 and at 276 ms (SE = 22) at P7 (**Figures 3B,C**). Peak-latency analyses revealed a shorter latency of the segmentation-related peak at Oz than the category-selective peak at the P7 or P8 electrode (**Figures 2A versus 2B** and **Figures 3A versus 3B,C**; confirmed by paired *t*-tests [P7: *t*(22) = 2.8; *p* = 0.009; P8: *t*(22) = 4.4; *p* < 0.001]. The precedence of the segmentation-related to the category-selective peak was confirmed even when both were studied at the P8 electrode [*t*(22) = -4.9; *p* < 0.001].

In conclusion, this experiment revealed that segmentation-related responses precede category-selective responses when evoked by texture-defined faces and houses. These results reject the hypothesis that categorization always precedes segmentation-related responses.

However, remaining questions are whether the category-selective responses reflect perceptual categorization or other, possibly cognitive processes, and whether the current results depend on stimulus parameters, such as low-level stimulus properties and presentation duration. Category-selective responses peaked at 304 ms, which is much later than the typical N170, the time at which face category-selective responses are usually maximal (e.g., Allison et al., 1999; Eimer, 2000a; Rossion et al., 2003; Itier and Taylor, 2004). This raises the question whether the textured objects at this peak were perceptually categorized into faces and houses. A control experiment confirmed that textured objects could be categorized into faces and houses (see below). This accords with previous behavioral and fMRI measurements showing categorization and face-specific FFA activity evoked by the same textured objects (Fahrenfort et al., 2012), which confirms that the faces are perceived as such. The current data furthermore suggest that the category-selective ERP peak reflects perceptual categorization, instead of for instance higher-level cognitive interpretation of the stimuli. Activity distributions indicate that texture-defined objects evoke a topographically highly similar response as photographic objects (**Figure 2**; row B versus C and D), but responses are weaker, and delayed by ∼200 ms. The fact that the responses are topographically similar (both in polarity and distribution), suggest that they reflect similar sources. Because both the category-selective and the preceding segmentation-related responses are present in occipital and parietal brain regions (**Figure 2**), it is more likely that they reflect perceptual categorization than cognitive processes. A reason for the delay in category-selective responses could be that they are evoked by second-order objects. Processing of second-order information, such as textured-defined objects, requires additional neural processing steps such as segmentation (for a review, see for example Baker and Mareschal, 2001). As such, category-selective responses based on second-order information might occur later than those based on first-order information (e.g., luminance differences present in photographic and line-drawing faces; Sagiv and Bentin, 2001). A related reason for the late category-selective responses could be that the texture-defined stimuli are not very naturalistic. Previous studies also show that category-selective responses can occur at varying timepoints: both EEG and MEG studies report face-selective responses at 170 ms or even earlier (e.g., Allison et al., 1999; Eimer, 2000b; Liu et al., 2002, 2009; Rossion et al., 2003; Itier and Taylor, 2004). On the other hand, presentation of faces for durations below the visible threshold also evoked category-selective responses occurring later in time than the classical N170 (Mitsudo et al., 2011). Furthermore, behavioral studies report different findings regarding categorization in relation to segmentation as well: some show that categorization precedes, while others show that it follows segmentation (Peterson, 1993, 1994; Grill-Spector and Kanwisher, 2005; Mack et al., 2008; Wyatte et al., 2012). It is unclear under which circumstances categorization is slow, following segmentation, or when it is fast, possibly even preceding segmentation. A contributing factor could be the optimality of stimulus presentation, which is affected by low-level stimulus properties and presentation duration.

Outside of the experimental setting the stimulus properties and presentation duration, based on which a face should be categorized vary as well. During a conversation with another person in the same room, a face is easily visible and continuously present. A soldier in the field on the other hand needs to rapidly categorize a camouflaged (and thus difficult to segment) face of the enemy in order to survive. Uncovering the effects of stimulus properties and presentation duration on the relation between subprocesses of face processing leads to a comprehensive picture of how the brain categorizes faces under varying circumstances. We therefore performed three more experiments that investigate the effects of stimulus properties and stimulus duration on the presence and timing of category-selective versus segmentation-related responses.

## Control Experiment: Behavioral Categorization of Textured Objects

To confirm that participants can categorize textured faces and houses, we performed a behavioral control run (13 healthy adults, average age 21.9 years, SD = 1.6; eight males). Participants performed a behavioral categorization task on the texture-defined objects as described in Experiment 1. Stimulus presentation consisted of a total of 21 stimuli per category. The experiment was self-paced to investigate the time participants required for categorization. Average response time was 823 ms, which is in the normal range for perceptual categorization tasks. Hits and FAs were compared to study behavioral performance. A distinction was made between segmentation and categorization hits and FA. Responses were defined as segmentation hit when a face or house was detected as object (i.e., face or house button presses for faces and houses, regardless of whether the category was correct) and FAs when a non-object was detected as object (i.e., face or house button presses for homogenous). Responses were defined as categorization hits when participants correctly detected a face as a face or a house as a house, and as FAs as those trials in which a house was detected as face or vice versa. Percentage correct was defined as the hit rate minus the FA rate.

Both faces and houses could be segmented and categorized above chance level, with close to 100% performance levels [more hits than FA; Segmentation: *t*(12) = 66; *p* < 0.001; percentage correct: 95.3% (SE = 0.94); Categorization: *t*(12) = 101; *p* < 0.001; percentage correct: 94.3% (SE = 1.44)]. These results confirm that textured objects could be categorized as faces and houses.

## Additional Experiments: Stimulus Properties Affect Presence and Timing of Category-Selective Responses

In three additional experiments we studied the effect of stimulus properties and presentation duration on the presence and timing of face category-selective responses. This section describes the methods and results per experiment, which are summarized in **Table 1**. The effect of stimulus properties was studied by comparing two types of stimuli: photographic and texture-defined stimuli, both containing faces and houses. Based on previous reports, suggesting a positive relation between stimulus visibility and timing of categorization (Kovács et al., 1995; Wyatte et al., 2012), we hypothesized that category-selective responses were faster when evoked by photographic than texture-defined faces and houses. For photographic and texture-defined stimuli, presentation duration of the stimuli was either long (800 ms, as in the main experiment) or short (92 ms). Because previous reports reveal categorization of photos of faces at 170 ms even after short presentation durations (Mitsudo et al., 2011), we did not expect an effect of presentation duration on category-selective responses in the photographic experiment. For texture-defined faces and houses, however, we did expect an effect of presentation duration on category-selective responses. Fast categorization is possible based on global outlines (Ahissar and Hochstein, 2004; Ahissar et al., 2009). However, the texture-defined objects are not very naturalistic and their global outlines might not provide sufficient category-specific information for fast categorization to occur. Presenting them for a short duration is even more suboptimal, which might further delay categorization processes.

### Experiment 2: Fast Categorization of Photographic Objects after Long Presentation Duration

The second experiment aimed to gain insight in the difference in processing speed under optimal (photographic objects) versus suboptimal conditions (texture-defined objects). Therefore, we studied the speed of category-selective responses evoked by photographic faces and houses presented for the same duration as in the first experiment (i.e., 800 ms).

#### Methods

Twenty-three healthy adults (average age 21.4 years, SE = 0.4; 12 males) participated in the study. Two other participants were excluded from the analyses, due to technical or human error during data acquisition.

**FIGURE 4:**
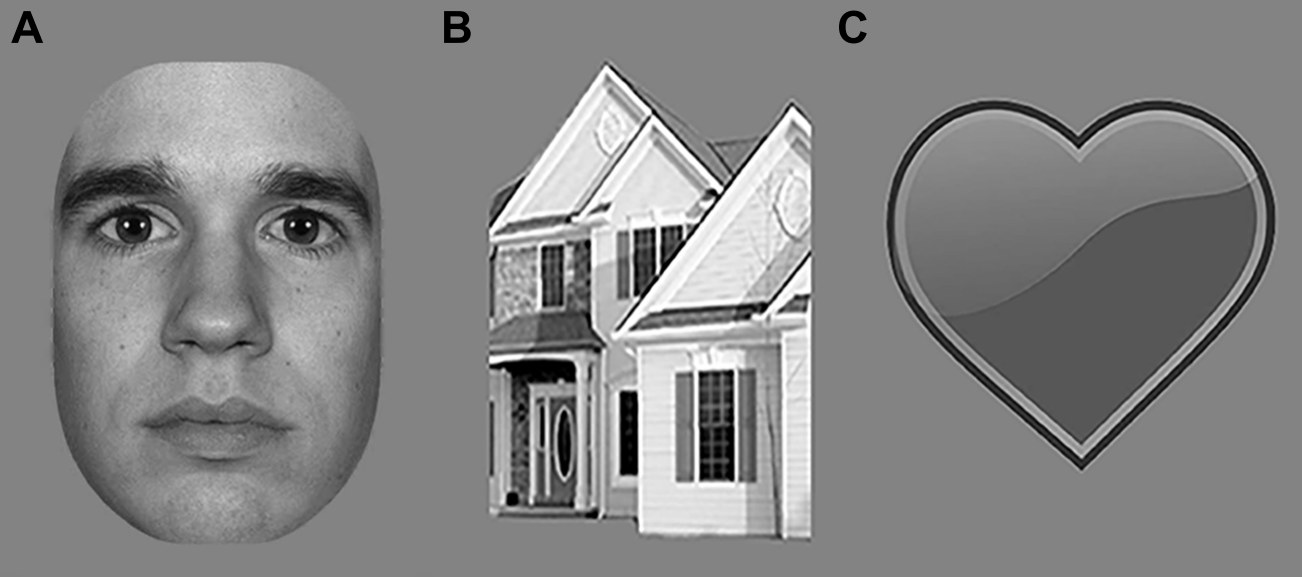
**Examples of stimuli presented to study categorization of photographic stimuli in Experiments 2 and 3; (A) Face; (B) House; (C) Heart (for task purposes)**.

Electroencephalography was acquired while participants viewed photographic stimuli of faces and houses (**Figure 4**). The MacBrain stimulus set^2^ provided face stimuli. We selected house stimuli and a cartoon heart from copyright-free internet sources. Using Photoshop, we cropped all stimuli, turned them into grayscale and matched them for size (11.5∘ × 10.5∘ of visual angle at a viewing distance of 57 cm). All faces had neutral expressions, to prevent effects of emotional expression content, and half were male. A total of 10 stimuli per condition were created. The experiment contained 60 stimuli per condition. 12.5% of the trials contained a heart-stimulus (see **Figure 4** for examples of face, house, and heart stimuli). The task of the participants was to press the spacebar when perceiving a heart. This task was the same as the one used in the main experiment. The stimulus sequence consisted of 800 ms object presentation, followed by a mask (scrambled version of the object; **Figure 4**) for 50 ms, and an inter-stimulus interval of 1600–2000 ms.

Analyses were equal to those described in the main experiment: cluster-based permutation tests detected category-selective responses in the EEG signal. If this was the case, ERP peak analyses revealed the latency at which the response was maximal. Furthermore, we compared category-selective responses between optimal (photographic objects) versus suboptimal conditions (texture-defined objects) using a paired samples *t*-test, to study differences in processing speed. Note that there is no clear-cut way of generating homogeneous versions of photographic objects. Although many experiments have contrasted intact objects with either block-scrambled or phase-scrambled objects (e.g., Malach et al., 1995; Grill-Spector et al., 2000), none of those are able to perfectly balance low-level physical stimulation between object and homogenous conditions as in classic segmentation studies (e.g., Lamme, 1995; Lamme et al., 1998). We therefore opted to not make homogenous versions of these stimuli, and we could consequently not make segmentation-contrasts for the photographic experiment.

#### Results and Discussion

Cluster-based permutation tests showed that photographic objects evoked category-selective responses (negative cluster, i.e., faces evoke more negative activity than house stimuli, between 68 and 500 ms after stimulus onset; **Figure 2C**). ERP peak latency analyses revealed that these responses were maximal at 165 ms (SE = 12.4; **Figures 3D,E**), represented in the N170 peak. These findings replicate multiple previous studies (e.g., Allison et al., 1999; Eimer, 2000a; Rossion et al., 2003; Itier and Taylor, 2004). Category-selective responses occurred at an earlier timepoint when evoked by photographic than texture-defined objects [*t*(22) = 5.2; *p* < 0.001], indicating that processes leading to categorization are faster under naturalistic than under artificial conditions.

### Experiment 3: Fast Categorization of Photographic Objects after Short Presentation Duration

In order to gain insight in the influence of presentation duration on the speed of processing of photographic objects, the third experiment studied the speed of category-selective responses evoked by photographic faces and houses presented for a shorter duration than in the first two experiments (i.e., 92 ms instead of 800 ms).

#### Methods

Eighteen healthy adults (average age 22.1 years, SE = 0.5; 11 males) participated in this experiment. One additional participant was excluded from analyses, due to human error during EEG acquisition. EEG recorded brain activity while participants viewed the same photographic faces and houses as described earlier, using the same procedure and presentation settings and the same analyses procedure. The only difference was that presentation duration of the stimuli was 92 instead of 800 ms.

#### Results and Discussion

Cluster-based permutation tests showed that photographic objects evoked category-selective responses (negative cluster, i.e., face evoke more negative activity than house stimuli, between 84 and 182 ms after stimulus onset; **Figure 2D**). ERP peak latency analyses revealed that this response was maximal at 156 ms (SE = 3.4; **Figures 3F,G**) after stimulus onset and represented in the N170 peak, very similar to the results obtained under longer duration. The results replicate previous findings (Mitsudo et al., 2011). Fast category-selective responses at the N170 peak resulting from photographic faces therefore do not seem to depend on stimulus duration.

### Experiment 4: Segmentation but No Categorization of Texture-Defined Objects after Short Presentation Duration

To gain further insight in the speed of processing of objects under highly artificial conditions (both texture-defined and short presentation duration), the fourth experiment studied the speed of category-selective and segmentation-related responses evoked by texture-defined faces, houses, and homogeneous stimuli presented for a shorter duration than in the main experiment (i.e., 92 ms instead of 800 ms). In the study of Fahrenfort et al. (2012), manipulating the visibility of object-percept was crucial to reveal category-selective responses in absence of segmentation-related responses. We used the same manipulation to study whether the speed of category-selective responses depends on the presence of segmentation-related responses.

#### Methods

Eighteen healthy adults (average age 22.1 years, SE = 0.5; 11 males) participated in this experiment. One additional participant was excluded from analyses, due to human error during EEG acquisition.

**FIGURE 5:**
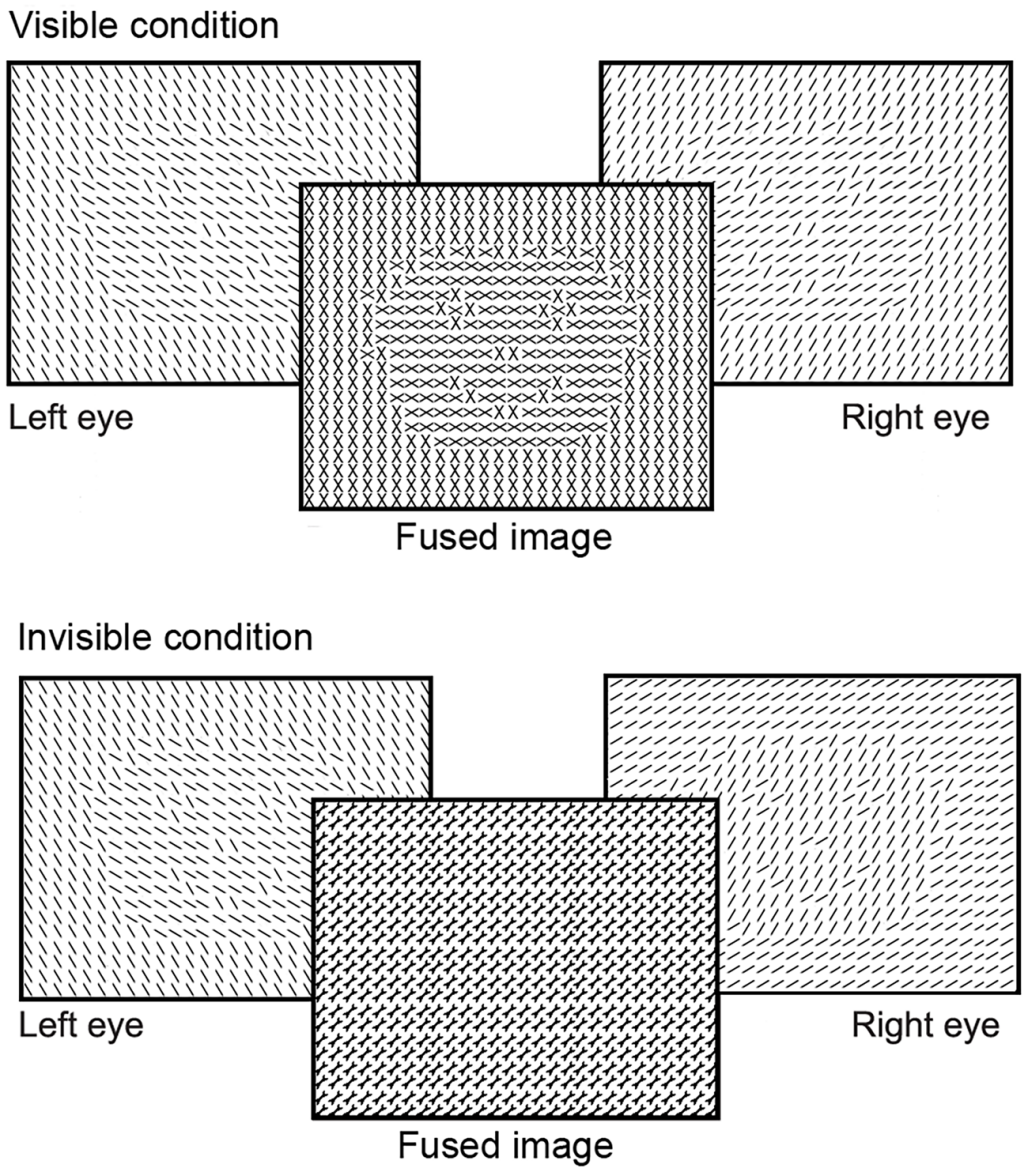
**Schematic versions of the stimuli presented to study segmentation and categorization of texture-defined objects in Experiment 4**. For clarity purposes, black lines instead of Gabor patches are depicted in this figure. Stimuli were presented to the left and right eye separately. Due to the short presentation time, stimuli for both eyes were fused into a visible or invisible image.

We recorded brain activity using EEG, while participants viewed texture-defined objects and homogeneous stimuli. As in the first experiment, stimuli were texture-defined faces or houses, or homogeneous textures. Stimulus construction and presentation differed from the first experiment, to keep stimulus parameters the same as in Fahrenfort et al. (2012). As in their study, we created a visible and invisible condition. Thus, we used a 3 (stimulus category: face, house, homogeneous) × 2 (stimulus visibility: visible, invisible) stimulus design. All stimuli contained a matrix of Gabor elements of specific orientations. Face- and house-categories were created using different orientations (22.5, 67.5, 112.5, or 157.5∘) for fore- and background. Gabor elements of the homogeneous stimuli had one of the four orientations per stimulus. To create visible and invisible conditions, we presented a stimulus to each of the eyes separately (referred to as dichoptic stimulation; Wolfe, 1983; Moutoussis and Zeki, 2002; Fahrenfort et al., 2012). Monocular presentation was achieved by having participants view a screen with a presentation rate of 120 Hz through shutter glasses, blocking the visual field to each eye alternatingly at a rate of 60 Hz. Consequently, each eye processed a different version of the same stimulus at a rate of 60 Hz. When different stimuli are presented to the left and the right eye for a short time, such as 92 ms in the current experiment, the brain fuses stimuli of the two eyes into a single percept rather than inducing binocular rivalry (Wolfe, 1983). Both eyes processed the same object, but the object contained differently oriented Gabor elements for the left and the right eye. Depending on the orientation of Gabors in the fore- and background, the dichoptic percept of the object was either visible or invisible (**Figure 5**). The figure was invisible when the orientation of the Gabors in the figure region of the object in the left eye was the same as that of the background of the object in the other eye (and vice versa). The figure was visible when the orientations between figure and background were different in the respective eyes (**Figure 5**). Thus, at a monocular level, stimuli in the visible and invisible condition were on average the same, but the dichoptic percept of stimuli was very different between conditions. Stimulus presentation contained two runs of 32 trials per stimulus condition (i.e., face, house, or homogeneous), resulting in a total of 192 randomly presented trials. Six percent of the stimuli consisted of a heart, composed of a similar matrix of Gabor elements as the texture-defined stimuli (**Figure 1A**). Each eye perceived half of the heart, such that participants perceived a complete heart when fusing the images in both eyes. Participants had to press the spacebar when a heart-stimulus was present. This task was the same as in the main experiment. The stimulus sequence consisted of object presentation for 92 ms, followed by a mask for 50 ms (field of Gabor elements with random orientation), and an inter-stimulus-interval for 1600–2000 ms (gray screen with fixation cross).

Analyses were equal to those described in the main experiment: cluster-based permutation tests detected segmentation-related and category-selective responses. If these responses were present, ERP peak analyses revealed the latency at which these responses were maximal.

#### Results and Discussion

Cluster-based permutation tests revealed that texture-defined objects presented for a short duration evoked segmentation-related responses in the visible condition (negative clusters: object > homogeneous; between 166 and 367 ms and between 408 and 500 ms after stimulus onset; **Figure 2E**). ERP peak analyses revealed that segmentation-related responses were maximal at 263 ms (SE = 15.6) after stimulus onset (**Figure 3H**). Stimuli in the invisible condition did not evoke segmentation-related activity (**Figures 2G and 3K**). Cluster-based permutation tests did not detect significant category-selective responses in the visible (**Figure 2F**) nor in the invisible condition (**Figure 2H**). Therefore, we performed no ERP peak analyses on category-selective responses (ERP depicted in **Figures 3I,J,L,M**).

The presence of segmentation-related responses in the visible, and absence in the invisible condition accords with previous research (Lamme et al., 1992; Zipser et al., 1996; Bach and Meigen, 1998; Fahrenfort et al., 2012). However, category-selective responses in the EEG were absent, as in Fahrenfort et al. (2012). This absence in the EEG responses could be due to multiple reasons. First, it is possible that objects did evoke category-selective responses but that EEG was not spatially sensitive enough to record it. Previous fMRI research recorded category-selective responses in the FFA evoked by texture-defined stimuli presented for a short duration (Fahrenfort et al., 2012). However, the N170 that one typically sees in EEG was argued to not only reflect activity in the FFA. Instead, it is likely to reflect a network response that includes the FFA, the occipital face area (OFA), and the posterior temporal sulcus (pSTS), and may even include distributed sources across the anterior fusiform gyrus together with activations in a parieto-temporal-occipital network (Rossion and Jacques, 2012). We will refer to these areas as the face-categorization network. It is possible that texture-defined faces presented for a short duration triggered the FFA, but not the full face-categorization network. In that case, EEG might not record category-selective responses even though they were present in some brain areas.

Another possibility is that some stimuli, such as those that lack naturalistic face properties, do not activate the face-categorization network if they are presented for a short duration. Theoretically, additional information that might fully activate the face-categorization network can be accumulated through recurrent connectivity from higher to lower areas in the visual hierarchy. However, this accumulation might require increased presentation duration (Ahissar et al., 2009). This can explain why long but not short presentation duration of texture-defined objects evoked category-selective responses. Finally, it is important to note that participants were not engaged in face-categorization during EEG acquisition. A lack of task-relevance might have interfered with effective processing of the stimulus. However, natural faces did evoke ERP responses related to categorization when participants performed the same task. This suggests that an absence of task-relevance does not (fully) explain the absence of categorization activity. Overall, these interpretations suggest that the combination of artificial stimulus properties and presentation duration did not activate the face-categorization network to an extent that EEG could record it.

## Summary and General Discussion

The current research studied the temporal relation between segmentation-related and face category-selective responses, measured using EEG. We contrasted brain activity evoked by texture-defined faces versus houses (category-selective responses) and by objects versus homogeneous stimuli (segmentation-related responses; **Figure 1C**), and subsequently compared the moments at which these responses were maximal. The hypothesis was that categorization- would precede segmentation-related responses. Results showed the opposite: segmentation-related preceded category-selective responses evoked by texture-defined objects. Crucially, however, category-selective responses in the EEG signal were much slower when evoked by texture-defined than by photographic stimuli. This suggests that the timing, and possibly temporal relation, of processes cannot be generalized from objects that are difficult to categorize to objects for which this is easier. Categorization of photographic faces might occur even before segmentation: category-selective responses evoked by photographic stimuli were detected earlier than segmentation-related responses typically occur (Lamme et al., 1992; Zipser et al., 1996; Bach and Meigen, 1998). The revealed precedence of segmentation-related to category-selective responses might thus be specific for objects presented under artificial or suboptimal conditions.

Previous neurocognitive and behavioral reports support the suggestion that processing speed depends on presentation settings. Fast categorization of photographic faces is in line with both EEG and MEG studies reporting face category-selective responses at 170 ms or even earlier (e.g., Allison et al., 1999; Eimer, 2000b; Liu et al., 2002, 2009; Rossion et al., 2003; Itier and Taylor, 2004). Such categorization is particularly fast for faces that can be categorized based on first-order information (e.g., luminance differences, as present in both photographic and line-drawing faces; Sagiv and Bentin, 2001). The current results suggest that category-selective responses across the entire face-processing network might be delayed when they depend on second-order information (e.g., differences in texture, as in the texture-defined objects) that requires an additional processing step before it can be evaluated (Baker and Mareschal, 2001). An important direction for future research is to explore the specific effects of presentation settings on the speed of category-selective responses.

The precedence of segmentation-related to category-selective responses during presentation of texture-defined objects is in line with previous results that segmentation or recurrent connectivity (which results in segmentation) leads to improved categorization of suboptimally presented objects (Koivisto et al., 2011; Wyatte et al., 2012). Furthermore, the conclusion that timing and possibly the temporal relation between segmentation-related and category-selective responses depend on presentation settings is in line with behavioral studies. Behavioral studies presenting objects with naturalistic settings (i.e., in accordance with the statistical regularities of the visual world we live in, such as familiar objects, clearly visible stimuli, long presentation duration, categorization-directed task) led to the conclusion that behavioral categorization precedes segmentation (Peterson, 1993, 1994; Grill-Spector and Kanwisher, 2005). On the contrary, studies presenting objects in artificial or less naturalistic settings (e.g., unfamiliar objects, distorted stimuli, short presentation duration, tasks in which categorization is irrelevant) led to the conclusion that categorization follows segmentation (Mack et al., 2008; Wyatte et al., 2012). Thus, the most optimal presentation context for fast categorization are naturalistic settings, and the speed of category-selective responses and possibly their temporal relation to segmentation-related responses, depends on the presentation context.

Altogether, one tentative interpretation of the current and previous results might be as follows: fast categorization is possible after natural stimulus presentation. Subsequently, the object is segmented which leads to behavioral categorization responses. When stimulation is suboptimal, as in artificial or cluttered scenes, fast categorization is not possible. Instead, slow categorization follows segmentation-related responses, ultimately leading to behavioral categorization of these stimuli as well.

These results are in line with the reverse hierarchy theory (RHT; Hochstein and Ahissar, 2002). The RHT proposes that categorization of clearly visible objects, such as photographic faces, is possible based on their global features in higher-level visual areas, such as the FFA. Global information reaches these higher areas via rapid feedforward connectivity. This matches the fast category-selective responses evoked by photographic faces and objects, even after short presentation duration (current study; Thorpe et al., 1996; Liu et al., 2002; Serre et al., 2007; Meeren et al., 2008). However, we also found that category-selective responses evoked by artificial texture-defined stimuli occurred at a much later timepoint than those evoked by photographic stimuli. This accords with the proposal of the RHT that if objects are difficult to categorize due to a low signal-to-noise ratio, feedback connectivity to lower areas provides additional information that can subsequently be used for categorization (Hochstein and Ahissar, 2002; Ahissar and Hochstein, 2004; Koivisto et al., 2011). Presumably, categorization based on these feedback processes requires repetitive or long presentation of stimuli (Ahissar et al., 2009). This accords with the occurrence of category-selective responses after long but not short presentation duration of texture-defined faces, and the precedence of segmentation, a result of feedback processes, to categorization for these stimuli.

Some of the methods differ between experiments in the current study, which restricts comparison of results across experiments and interpretation of effects. For example, photographic and texture-defined objects are very different from each other, for instance in ecological validity. Because the texture-defined objects evoked later category-selective responses than at the typically observed N170 peak, it is important to consider whether both responses reflect the same underlying processes. As discussed above, it is likely that additional processes are involved in categorization of each of the objects, such as recurrent connectivity for the texture-defined objects. The measured category-selective responses thus might not reflect the results of the same flow of information in the brain. However, the category-selective responses seem to reflect perceptual categorization, instead of for instance cognitive processes. Activity distributions (**Figure 2**) show that textured and photographic objects result in the same topographical distribution across occipital and parietal areas, but that the category-selective responses evoked by textured objects is weaker and delayed. The preceding process, being segmentation-related responses, also evokes occipital activity. Therefore, it is more likely that the category-selective responses evoked by textured objects reflect perceptual categorization, at least closely linked to the N170 category-selective responses evoked by photographic objects, instead of cognitive processes. Furthermore, although segmentation-related and category-selective responses were studied using separate stimulus-contrasts, the objects in the segmentation contrast did contain specific categories. As a result, a more general and crude type of category extraction (that of differentiating between faces and homogenous screens and/or that of differentiating between houses and homogenous screens) might have contributed to the segmentation-related responses. However, the results show that the onset of the segmentation contrast preceded the moment in time at which specific categorization responses between faces and houses occurred. Therefore, any contribution of ‘crude categorization’ to segmentation would not be category specific in the classic sense of one category versus another category. Moreover, the contribution of other potential processes, such as object detection, cannot be isolated from segmentation-related activity in the current study. The distinction between segmentation and detection is not one that can be made based on contrasts alone, as it also depends on the psychological and physiological definitions of these processes. One interpretation is that on a neural level, object detection reflects boundary detection, which like some categorization responses can already occur during the first feedforward sweep, while on a psychological level, explicit object detection reflects segmentation processes, requiring recurrent interactions in visual cortex (e.g., see Fahrenfort et al., 2007 for an experiment in which these two processes are isolated and related to behavioral detection). Finally, the results of the current study might be specific to processing of faces and not be generalized to other object categories. Faces are often considered a special category, because they evoke activity in a specific brain area (the FFA) and humans are sensitive to even small changes in a face (McKone and Robbins, 2011). Future research should reveal whether category-selective responses are similar in texture-defined faces and other objects, and whether segmentation-related precede category-selective responses for other objects as well. Another important avenue for research is the question to which extent the N170 reflects activation in the FFA, or in a broad face-activation network including the OFA and pSTS that depends more strongly on segmentation than the initial FFA response.

Nevertheless, the current findings have important implications for future research, by promoting that one should take into account the role of segmentation in face categorization. This is the case for research into typical face processing, but is particularly valuable for research into atypical face processing or its development. An unresolved question in these fields is which subprocesses contribute to development of typical and atypical face processing. The successful separation of segmentation-related and category-selective responses provides exciting opportunities for studying the contribution of these subprocesses to face processing in various populations. As the relation between subprocesses depends on the optimality of stimulus presentation, one should differentiate between the typicality of face categorization during optimal versus suboptimal presentation. This is especially important when studying populations that have difficulties in face processing during optimal presentation, such as persons with Autism Spectrum Disorder (Golarai et al., 2006), or that often come across situations of suboptimal face presentation, such as soldiers that need to detect the camouflaged enemy. The ability to segment an object seems crucial for successful categorization in such cases. Previous research reported that segmentation could be trained through perceptual learning methods in both adults and children, which improves both behavioral and neural reflections of segmentation (Censor et al., 2009; Gervan et al., 2012). This provides exciting possibilities to improve categorization even in suboptimal conditions.

## Conclusion

The current study shows that although there is increasing evidence that categorization often results from feedforward processing (Thorpe et al., 1996; Riesenhuber and Poggio, 2000; Liu et al., 2002; Serre et al., 2007), while segmentation requires recurrent processing (Lamme and Roelfsema, 2000; Appelbaum et al., 2006; Fahrenfort et al., 2012), segmentation nevertheless precedes category-selective responses when objects lack low-level image properties to aid in fast categorization. Our results increase the understanding of the inter-relation between segmentation and categorization, and the speed of category-selective responses under varying circumstances.

## Conflict of Interest Statement

None of the authors or related institutions received payment or service from a third party for any aspect of the presented work. None of the authors have financial relationships with entities that could be perceived to influence, or that give the appearance of potentially influencing, the presented work. The authors declare that the research was conducted in the absence of any commercial or financial relationships that could be construed as a potential conflict of interest.

## Footnotes

1 Fieldtrip is an open source Matlab toolbox for EEG and MEG analysis; (Oostenveld et al., 2011).

2 Development of the MacBrain Face Stimulus Set was overseen by Nim Tottenham and supported by the John D. and Catherine T. MacArthur Foundation Research Network on Early Experience and Brain Development. Please contact Nim Tottenham at tott0006@tc.umn.edu for information concerning the stimulus set.
